# Sleep disturbances among young adult dual users of cigarettes and e-cigarettes: Analysis of the 2020 National Health Interview Survey

**DOI:** 10.1371/journal.pone.0320748

**Published:** 2025-03-25

**Authors:** Laxmi Poudel, Amrit Baral, Alireza Abdshah, Kyle Grealis, Anurag Aka, Shreedhar Paudel, Denise C. Vidot

**Affiliations:** 1 South Florida Integrative Medicine, Miami, Florida, United States of America; 2 University of Miami Miller School of Medicine, Miami, Florida, United States of America; 3 University of Miami School of Nursing and Health Sciences, Miami, Florida, United States of America; 4 Massachusetts General Hospital Brigham, Boston, Massachusetts, United States of America; 5 Harvard Medical School, Boston, Massachusetts, United States of America; University of Foggia: Universita degli Studi di Foggia, ITALY

## Abstract

**Background:**

The increasing use of conventional cigarettes and e-cigarettes among young adults (18-35 years) in the US raises significant health concerns, including impacts on sleep. While smoking’s adverse health effects are well-documented, the combined effects of conventional cigarette and e-cigarette use on sleep remain under explored, particularly in young adults. This study investigates the association between dual cigarette and e-cigarette use and sleep outcomes in a nationally representative US sample.

**Methods:**

We utilized self-reported data from the 2020 National Health Interview Survey (NHIS) on young adults (N = 6128, Unweighted). Descriptive statistics and chi-squared tests and t-tests, where appropriate, compared socio-demographic, clinical, behavioral, and sleep-related characteristics by conventional cigarette/e-cigarette use status. Multinomial logistic regression estimated the odds of reporting short (<7 hours) or long sleep ( ≥ 9 hours) compared to normal sleep (7-8 hours) across different smoking categories.

**Result:**

Of the total sample, 51.0% were females, mean age: 26.6 years (SD = 4.8). Cigarette smokers were the oldest (mean age 29.2 years), while e-cigarette users were the youngest (mean age 24.7 years) (p < .0001). Poor or long sleep was reported by 72.8% of cigarette smokers, 69.4% of e-cigarette users, and 71.9% of dual users (p < 0.001). Trouble falling asleep daily, or most days was reported by 49.9% of cigarette smokers, 63.6% of e-cigarette users, and 58.5% of dual users (p < 0.001). Difficulty staying asleep daily for most days was reported by 38.0% of cigarette smokers, 45.0% of e-cigarette users, and 44.6% of dual users (p < 0.0001). Furthermore, 13.7% of cigarette smokers, 5.9% of e-cigarette users, and 12.3% of dual users reported never waking up well-rested (p < 0.001). Multinomial logistic regression revealed that cigarette-only users (aOR:1.40, 95%CI:1.06-1.85), e-cigarette users (aOR:1.32, 95%CI:1.06-1.66), and dual users (aOR:1.81, 95%CI:1.46-2.24) had 40%, 32%, and 81% higher odds, respectively, of having poor sleep compared to non-users.

**Conclusion:**

Cigarette and e-cigarette use is associated with poor sleep patterns and quality, with dual users having the greatest odds of having poor sleep outcomes among young adults.

## Introduction

Poor sleep health is a major public health concern. Recent evidence suggests that sleep problems often coexist with and contribute to various health conditions, both physical and mental [1]. Sleep involves both the duration and the quality of rest as crucial aspects [2]. The duration of sleep, referring to the total hours of sleep acquired in a day, significantly impacts the overall health and well-being of young adults [3]. Short sleep duration, commonly termed inadequate sleep, is a public health issue linked to higher risks of adverse health conditions including obesity, diabetes mellitus, cardiovascular disease, depression, and emotional regulation issues [4]. Sleep quality defined as “an individual’s self-satisfaction with all aspects of the sleep experience has emerged as a crucial determinant of physical and mental well-being [5]. The pursuit of high-quality sleep has prompted significant advancements in the understanding of sleep patterns and their impact on various health parameters. Since the discovery of brain activities during sleep in the 1970s and 1980s, the importance of sleep has been progressively underscored, leading to the identification of numerous sleep disorders by 2005 [6]. As science and technology have advanced, the intricate interplay between sleep and health conditions has been rigorously explored, revealing links between sleep and various health conditions [7–9].

Meanwhile, tobacco use has persistently posed significant health risks, with smoking standing as the foremost cause of preventable diseases, disabilities, and deaths in the United States (US) [10]. In the US, the prevalence of cigarette smoking remains a substantial public health concern, despite efforts to curtail its impact [11]. According to 2021 data, an estimated 11.5% of adults (approximately 28.3 million) were current cigarette smokers, with 13.1% being men and 10.1% women. Every day, about 1,600 young people under the age of 18 years smoke their first cigarette, and 235 individuals begin smoking cigarettes daily. Moreover,16 million people in the US live with at least one disease caused by smoking, and a staggering 58 million nonsmoking Americans are exposed to secondhand smoke [12,13].

The consequences of smoking extend across various domains of health, including cancer, cardiovascular disorders, respiratory, and reproductive system complications, cognitive functions, and even sleep functions [14–16]. As society evolves, new avenues of nicotine consumption have emerged, most notably the rise of electronic cigarettes (e-cigarettes). By 2021, the number of e-cigarette users had reached 10.8 million among US adults, with a noteworthy finding that 15% of these users had never smoked conventional cigarettes. The prevalence of current e-cigarette use was most pronounced among individuals aged 18 to 24 years, accounting for 9.2% of this age group (approximately 2.8 million users). Strikingly, more than half (51.2%) of all current e-cigarette users were younger than 35 years old [17,18].

Despite the decline in conventional cigarette use in recent years, e-cigarette usage has been on the rise, and this shift is particularly noticeable among specific demographics. In light of this evolving landscape of tobacco consumption habits, the phenomenon of dual-use—the simultaneous use of conventional cigarettes and e-cigarettes—has emerged as an area of research interest. However, this realm remains relatively unexplored, with limited research focusing on the associations between dual tobacco use and sleep outcomes. Despite numerous studies suggesting potential links between tobacco usage and disrupted sleep patterns, few have investigated the impact of combined cigarette and e-cigarette consumption on sleep quality, quantity, and overall sleep parameters [16–23]. According to the Centers for Disease Control and Prevention’s National Center for Health Statistics (CDC NCHS), adults aged 18–24 and 25–44 are more likely to be dual users of e-cigarettes and cigarettes compared to adults aged 45 and over, with e-cigarette use being highest among adults aged 18–24 [24]. This demographic trend underscores the importance of studying this population,

This study aims to address this critical gap by delving into the intricate relationship between dual cigarette and e-cigarette usage and its potential effects on sleep patterns and quality. Leveraging data from the National Health Interview Survey (NHIS), a nationally representative sample of the U.S. population, our research endeavors to elucidate the nuanced connections between tobacco consumption and sleep outcomes.

## Methods

### 2.1. Data source and study sample

We utilized the 2020 National Health Interview Survey (NHIS) data (N = 6128, unweighted) for young adults (18-35 years old) to estimate the association between dual use of conventional cigarettes and e-cigarettes with sleep outcomes. The NHIS is a population-based annual household survey of the health and healthcare of the U.S. non-institutionalized, civilian population administered by the U.S. Centers for Disease Control and Prevention (CDC). The survey involves face-to-face household interviews, conducted by trained NCHS interviewers, to collect data from selected respondents completing the NHIS instrument. The sampling design is multistage and stratified to ensure a nationally representative dataset for the US civilian noninstitutionalized population, covering all 50 states and the District of Columbia. Computer-assisted data collection is used to check data quality and maintain questionnaire consistency during interviews [25,26]. We accessed the dataset on October 18, 2023, from the CDC’s National Center for Health Statistics website (https://www.cdc.gov/nchs/nhis/index.htm. The data used in this study were publicly available, fully de-identified, and did not involve interactions with living human subjects or access to identifiable information. All methods were performed in accordance with the relevant guidelines and regulations. Each household selected for NHIS participation receives an advance letter a week before the interview period, explaining the survey’s purpose, duration, and voluntary nature, and assuring confidentiality. Upon arrival, the interviewer provides another copy of the letter and obtains verbal consent from respondents [27].

### 2.2. Study measures

#### 2.2.1. E-cigarette and Conventional (combustible) cigarette use status.

Participants were asked about their current use of e-cigarettes and combustible cigarettes, with response options including “every day,” “some days,” and “not at all.” Their responses were combined into four categories to identify usage patterns: (a) Non-users: Individuals who reported not using e-cigarettes or smoking combustible cigarettes at all. (b) Exclusive e-cigarette users: Those who reported using e-cigarettes daily or occasionally but did not smoke combustible cigarettes. (c) Exclusive combustible cigarette smokers: Participants reported smoking combustible cigarettes daily or occasionally but did not use e-cigarettes. (d) Dual product users: Respondents who reported using both e-cigarettes and smoking combustible cigarettes daily or occasionally.

#### 2.2.2. Sleep duration, quality of sleep variables, and sleep medication use.

NHIS surveys collect data on the hours of sleep of individuals by asking, *“On average, how many hours of sleep do you get in 24 hours?”*. Based on a prior published study [28,29], we categorized the duration of sleep into three levels: short sleep (<7 hours), normal sleep (7-8 hours), and long sleep (≥9 hours). Similarly, NHIS surveys gather variables to assess the quality of: (1). “Trouble falling asleep”, (2). “Trouble staying asleep”, and (3). “Waking up feeling well-rested”, derived from the questions: *“During the past 30 days, how often did you have trouble falling asleep?”, “During the past 30 days, how often did you have trouble staying asleep” and “During the past 30 days, how often did you wake up feeling well-rested?”* respectively. The responses to these questions were ‘Never’, ‘Somedays’, ‘Most days’, and ‘Every day’.

Sleep medication use was assessed using the question: *“During the past 30 days how often did you take any medication to help you fall asleep or stay asleep? Include both prescribed and over-the-counter medications.* Response options were never, some days, most days, or every day. This variable was re-coded to create a binary (yes/no) variable.

#### 2.2.3. Covariates.

Sociodemographic variables included were the participant’s age, sex (male and female), race/ethnicity (Hispanic, non-Hispanic White, non-Hispanic Black, non-Hispanic Asian, and other), annual family income level, and education. Body mass index, general health status, and alcohol use were also included as potentially important factors that can affect sleep among young adults [3]. Additionally, we included self-reported lifetime asthma diagnosis and respiratory disorders (COPD, emphysema, or chronic bronchitis), since respiratory conditions are associated with sleep and well as smoking [30,31].

### 2.3. Statistical analysis

Descriptive statistics and chi-squared tests as well as t-tests where appropriate were used to examine the differences in the distribution of the socio-demographic, clinical, behavioral, and sleep-associated characteristics among US young adults by smoking status (Tables 1 and 2).

**Table 1:** Sociodemographic, behavioral, and clinical characteristics (weighted proportions) of U.S young (18-35-years-old) adults by cigarette and e-cigarette use status, 2020, NHIS.

		Cigarette and E-cigarette Use Status				
	Overall	Neither	Cigarette only	E-cigarette only	Dual use	p-value
*Unweighted sample (N)*	6,128	3,774	494	946	914	
	**Weighted proportions (%)**					
		61.4	7.8	16.3	14.5	
**Age (years)**						<.0001
Mean (SD)	26.6 (4.8)	26.4 (4.9)	29.2(3.8)	24.7(4.7)	28.2(4.2)	
**Sex**						<.0001
Female	51.0	56.7	44.0	39.8	35.9	
Male	49.0	43.3	56.0	60.2	64.1	
**Race/ethnicity**						<.0001
Hispanic	21.2	23.3	17.0	22.4	12.8	
NHW	55.8	50.6	58.7	60.2	70.7	
NHB/AA	12.8	15.1	14.5	8.8	6.9	
NHA	6.8	8.	5.8	5.3	3.9	
Other	3.4	2.0	3.8	3.3	5.7	
**BMI**						<.001
Underweight	2.2	2.6	2.1	1.6	1.6	
Healthy weight	39.9	41.6	30.8	41.7	35.7	
Overweight	29.2	28.6	34.1	29.4	28.6	
Obese	28.7	27.2	33.0	27.3	34.1	
**General Health Status**						<.0001
Excellent	35.1	39.4	26.0	33.7	23.0	
Very good	37.5	36.1	38.1	40.3	39.9	
Good	21.7	21.7	28.4	19.7	27.2	
Fair	5.0	4.3	6.9	3.8	8.2	
Poor	0.7	0.5	0.6	0.5	1.7	
**Income level**						<.01
$0-$34,999	22.4	21.6	28.5	20.6	24.5	
$35,000-$49,999	13.1	12.4	15.3	12.3	15.6	
$50,000-$74,000	18.9	18.7	20.9	18.4	19.3	
$75,000-$99,999	14.7	14.8	12.4	14.8	15.1	
≥$100,000	30.9	32.5	22.9	33.9	25.5	
**Education level**						<.0001
<HS diploma	9.2	8.6	17.9	6.8	9.6	
GED/HS diploma	29.1	26.9	37.5	26.5	35.9	
Some college/college degree	53.8	54.8	38.6	60.4	50.8	
Master/Doctoral	7.9	9.7	6.0	6.3	3.7	
**Alcohol use status**						<.0001
Current: heavy	6.0	2.5	9.6	8.3	16.5	
Current: rare to moderate	68.2	63.1	70.7	82.6	72.7	
Former	8.5	8.9	12.5	4.4	9.3	
Abstainer	17.3	25.5	7.2	4.7	1.5	
**Asthma**						0.013
Yes	17.6	16.2	16.4	20.5	21.0	
No	82.4	83.8	83.6	99.5	79.0	
Chronic Respiratory Disorders						0.022
Yes	1.2	0.8	0.8	0.9	3.9	
No	98.8	99.2	99.2	99.1	96.1	

***Abbreviations:*** NHIS- National Health Interview Survey; BMI- Body Mass Index; NHW- Non-Hispanic White; NHB- Non-Hispanic Black; NHA-Non-Hispanic Asian.

* p-value is calculated using chi-squared test/student’s t-test where appropriate.

Respiratory diseases include chronic obstructive pulmonary diseases.

**Table 2:** Sleep characteristics (weighted proportions) of U.S. young adults (18-35-years-old) by cigarette and e-cigarette use status, 2020, NHIS.

	Overall	Neither	Cigarette only	E-cigarette only	Dual use	p-value
*Unweighted sample (N)*	6128	3774	494	946	914	
	**Weighted proportions (%)**					
**Sleep duration**						<.0001
Long ( ≥ 9 hours)	44.4	47.2	42.7	43.2	35.5	
Normal (7-8 hours)	30.6	31.5	27.2	30.6	28.1	
Poor ( < 7 hours)	25.0	21.3	30.1	26.2	36.4	
**Sleep medication use**						<.0001
No	84.2	87.1	87.7	79.0	75.7	
Yes	15.8	12.9	12.3	21.0	24.3	
**Wake up well rested**						<.0001
Everyday	11.0	13.8	12.6	4.9	5.2	
Most days	41.0	42.3	36.4	43.0	35.8	
Some days	39.3	35.9	37.3	46.2	46.7	
Never	8.7	8.0	13.7	5.9	12.3	
**Trouble falling asleep**						<.0001
Never	4.9	3.3	6.6	5.3	10.6	
Every day	11.4	9.1	9.8	16.7	16.4	
Most days	42.3	41.5	40.1	46.9	42.1	
Some days	41.4	46.1	43.5	31.1	30.9	
**Trouble staying asleep**						<.0001
Never	55.7	59.3	54.6	51.6	45.7	
Every day	31.2	30.2	31.8	34.3	32.2	
Most days	8.3	6.9	6.2	10.7	12.4	
Some days	4.8	3.6	7.4	3.4	9.7	

***Abbreviations:*** NHIS- National Health Interview Survey.

Multinomial logistic regression analysis was conducted to estimate the odds ratios and 95% confidence intervals for the association between usage of cigarette-only, e-cigarette-only, or both and sleep duration with neither use as a reference category, adjusting for potential confounders and covariates (Table 3).

**Table 3:** Adjusted Odds ratios with 95% CI for sleep duration and cigarette/e-cigarette use status among US young adults (18-35-years-old) using multinomial logistic regression analysis, 2020 NHIS.

Variables	Poor sleep vs. Normal sleep		Long sleep vs. Normal sleep	
	^a^Model 1A: AOR with 95%CI	^b^Model 1B: AOR with 95%CI	^a^Model 2A: AOR with 95%CI	^b^Model 2B: AOR with 95%CI
Cigarette only	1.56 (1.15-2.12)	1.40 (1.06-1.85)	1.17 (0.90-1.57)	1.62 (1.00-2.61)
E-cigarette only	1.37 (1.10-1.75)	1.32 (1.06-1.66)	0.90 (0.72-1.13)	1.34 (0.94-1.89)
Dual use	2.0 (1.58-2.52)	1.81 (1.46-2.24)	0.95 (0.76-1.20)	1.16 (0.75-1.82)
Neither (Ref)	–	–	–	–

***Abbreviations:*** NHIS- National Health Interview Survey; AOR- Adjusted odds ratio.

^a^:Adjusted for socio-demographic variables (age, sex, race/ethnicity, education).

^b^:Adjusted for socio-demographic variables, clinical (body mass index, sleep medication use, general health status), behavioral variables (alcohol use), and respiratory disorders (Chronic obstructive pulmonary disease and Asthma).

We incorporated appropriate survey sampling weights provided by NHIS in all analyses. All statistical tests were two-tailed, with an alpha less than 0.05 considered statistically significant. SAS version 9.4 (SAS Institute, Inc., Cary, NC, USA) was used for statistical analysis. All the participants had provided CDC with informed consent at registration and data were de-identified.

## Results

Among the 6128 (unweighted) young adults (18-35-years-olds) included in this study 51% were female, the majority were non-Hispanic White (55.8%) followed by Hispanic (21.2%) (Table 1). The mean age of the overall sample was 26.6 (SD = 4.8), the majority were in the healthy weight category (39.9%), and 72.6% reported excellent or very good general health status. Regarding income, 30.9% were in the highest income bracket (≥$100,000). More than half (53.8%) of the respondents had some college education or college degree and 68.2% reported current (rare to moderate) use of alcohol. A larger proportion of dual users (16.5%) were also current: heavy alcohol consumers. In contrast, more non-smokers (25.5%) were alcohol abstainers (25.5%).

Of the total sample, 7.8% were combustible (conventional) cigarette-only users, 16.3% used e-cigarette-only users, and 14.5% were dual users. Cigarette-only smokers were the oldest with a mean age of 29.2 (SD = 3.8) years old, followed by dual users (28.2, SD = 4.2), non-smokers (26.4, SD = 4.9), and e-cigarette users (24.7, SD = 4.7) (P < .0001). 56% of cigarette smokers, 60.2% of e-cigarette smokers, and 64.1% of dual users were male (P < 0.001). 67.1% of smokers, 56.7% of e-cigarette users, and 62.7% of dual users were overweight and obese (P < 0.001). 56.2% of cigarette smokers, 67.1% of e-cigarette smokers, and 59.9% of dual smokers had an average income above 50,000 US dollars (P < 0.01). There was a higher prevalence of lifetime diagnosis of asthma among dual users (21.0%) followed by e-cigarette-only users (20.5%), cigarette-only users (16.4%), and non-users (16.2%, p = 0.013). Likewise, chronic respiratory diseases were more prevalent among dual users (3.9%) as compared to e-cigarette-only users (0.9%), cigarette-only users (0.8%), and non-users (0.8%, p = 0.022).

**Fig 1:**
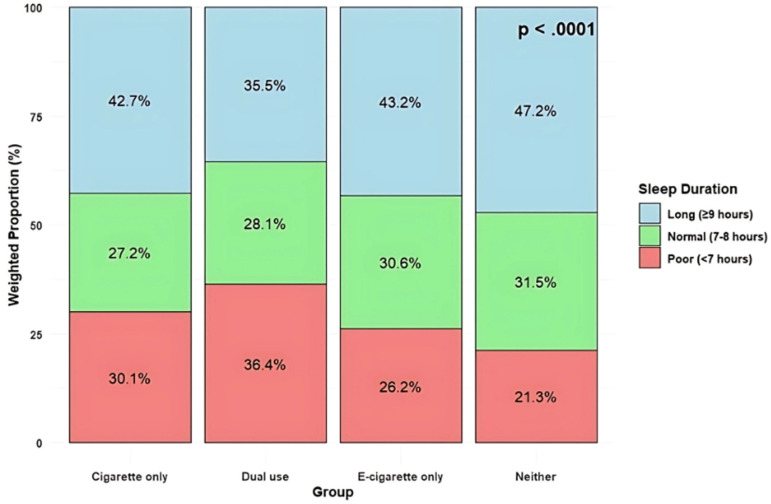
Weighted proportions of sleep duration by cigarette/e-cigarette use status among U.S. young adults (18-35-years-old), 2020 NHIS (N = 6,128).

Table 2 depicts the sleep characteristics of US young adults by cigarette and e-cigarette use status. There was a significant difference in sleep duration among the different user groups (p <  0.0001). Overall, 44.4% of young adults reported long sleep durations (≥9 hours), with the highest proportion in the “Neither” group (47.2%) and the lowest in the “Dual use” group (35.5%). Normal sleep duration (7-8 hours) was reported by 30.6% overall, with the “Neither” group again having the highest proportion (31.5%) and the “Cigarette only” group the lowest (27.2%). Poor sleep duration (<7 hours) was most prevalent in the “Dual use” group (36.4%) and least common in the “Neither” group (21.3%) (Fig 1). The use of sleep medication also showed significant variation (p <  0.0001). The majority of participants (84.2%) did not use sleep medication, with the highest non-use reported in the “Cigarette only” group (87.7%) and the lowest in the “Dual use” group (75.7%). Conversely, the “Dual use” group had the highest proportion of sleep medication use (24.3%), while the “Cigarette only” group had the lowest (12.3%). The frequency of waking up well-rested varied significantly among the groups (p <  0.0001). Overall, only 11.0% of participants reported feeling well-rested every day, with the highest percentage in the “Neither” group (13.8%) and the lowest in the “Dual use” group (5.2%). Most days feeling well-rested were reported by 41.0% overall, with the highest in the “E-cigarette only” group (43.0%) and the lowest in the “Dual use” group (35.8%). The “Dual use” group also had the highest proportion of participants never feeling well-rested (12.3%), compared to 8.0% in the “Neither” group. Significant differences were found in trouble falling asleep among the groups (p <  0.0001). A small proportion (4.9%) reported never having trouble falling asleep, with the “Neither” group at the lowest (3.3%) and the “Dual use” group at the highest (10.6%). Trouble falling asleep every day was most common in the “E-cigarette only” group (16.7%) and least common in the “Neither” group (9.1%). Trouble falling asleep most days was reported by 42.3% overall, with the highest in the “E-cigarette only” group (46.9%) and the lowest in the “Neither” group (41.5%). There were significant differences in trouble staying asleep among the groups (p <  0.0001). More than half of the participants (55.7%) reported never having trouble staying asleep, with the highest proportion in the “Neither” group (59.3%) and the lowest in the “Dual use” group (45.7%). Trouble staying asleep every day was most common in the “E-cigarette only” group (34.3%) and least common in the “Neither” group (30.2%). The “Dual use” group had the highest proportion of participants experiencing trouble staying asleep most days (12.4%), while the “Cigarette only” group had the lowest (6.2%).

Table 3 the multinomial logistic regression analysis examined the association between cigarette/e-cigarette use and sleep duration among U.S. young adults (18-35 years old) using data from the 2020 National Health Interview Survey (NHIS). The reference group for all comparisons was individuals who neither smoked cigarettes nor used e-cigarettes. The results are presented as adjusted odds ratios (AOR) with 95% confidence intervals. For poor sleep vs. normal sleep, after adjusting for socio-demographic variables (Model 1A), the AOR was 1.56 (95% CI: 1.15-2.12), and after further adjustment for clinical and behavioral variables (Model 1B), the AOR was 1.40 (95% CI: 1.06-1.85). E-cigarette-only users had AORs were 1.37 (95% CI: 1.10-1.75) and 1.32 (95% CI: 1.06-1.66) in Models 1A and 1B, respectively. Dual users exhibited the highest odds of poor sleep with AORs 2.00 (95% CI: 1.58-2.52) and 1.81 (95% CI: 1.46-2.24) in Models 1A and 1B, respectively.

In the analysis of long sleep vs. normal sleep (Table 3), cigarette-only users after adjusting for socio-demographic variables (Model 2A), the AOR was 1.17 (95% CI: 0.90-1.57), and after further adjustment for clinical and behavioral variables (Model 2B), the AOR was 1.62 (95% CI: 1.00-2.61). E-cigarette-only users had AORs of 0.90 (95% CI: 0.72-1.13) and 1.34 (95% CI: 0.94-1.89) in Models 2A and 2B, respectively. Dual users had an AOR of 0.95 (95% CI: 0.76-1.20) and 1.16 (95% CI: 0.75-1.82) in Models 2A and 2B, respectively.

## Discussion

This study aimed to explore the association between the dual use of conventional cigarettes and e-cigarettes and sleep outcomes among young adults aged 18-35 using a nationally representative dataset. The results indicate that dual users had significantly higher odds of experiencing poor sleep outcomes. Specifically, in the fully adjusted model when adjusting for socio-demographic, clinical, and behavioral variables, dual users had 81% greater odds of having poor sleep, cigarette-only users had 40% greater odds, and e-cigarette-only users had 32% greater odds when compared to non-users. Furthermore, comparisons of sleep duration by cigarette/e-cigarette use status found that the dual users had the lowest proportion of respondents reporting long sleep duration (35.5%) and the highest proportion reporting poor sleep (36.4%). Analysis of sleep quality revealed that cigarette smokers, e-cigarette users, and dual users all reported significant differences in sleep quality with dual users consistently reporting the worst outcomes, suggesting that the combined use of both products exacerbates sleep disturbances.

The observed trend of lower sleep quality in this study is consistent with previous research on dual users. An analysis of the Behavioral Risk Factor Surveillance System in young adults revealed that dual users were at a higher risk of reporting poor sleep duration compared to non-users and e-cigarette-only users [3]. This corroborates our findings, which also demonstrate that dual users experience significantly poorer sleep outcomes. Utilizing another nationally representative dataset, our study confirms these associations and expands the understanding of sleep health in this demographic by providing additional insights into other critical sleep-related outcomes. Specifically, we observed that dual users not only had poorer sleep duration but also reported higher levels of difficulty falling and staying asleep, consistent with previous studies that identified dual usage as a factor in increasing sleep latency [32]. Our study also accounted for other sleep outcomes associated with dual use, finding that dual users exhibited greater usage of sleep medications. It has previously been noted that e-cigarette users report significantly higher rates of usage for sleep medications compared to combustible cigarette smoking, however, the trend has not been explored in dual users [23]. Given that sleep medication usage is typically an indicator of poor sleep, rates of sleep medication use amongst dual users agree with other significant findings related to proportionally worse sleep outcomes in dual users. There may also be a biological mechanism with sleep medications that warrants further investigation.

The mechanistic actions underlying the poorer sleep outcomes observed in dual users can be attributed to several factors. Nicotine, a well-known stimulant, plays a significant role in sleep disruption [33]. Higher nicotine concentrations in dual users suggest a synergistic and possible dose-response effect that can contribute to this effect. Moreover, analyses from both American and Korean datasets have suggested that dual users exhibit higher rates of nicotine dependence [34,35]. This suggests that dual use could contribute to nocturnal awakenings due to cravings, along with higher nicotine use before sleeping. Furthermore, dual users are at an increased risk of developing pulmonary diseases, which are associated with chronic cough and other pulmonary issues that disrupt sleep [32]. Specifically, a study in Korea using the STOP-Bang score has found that both dual users and cigarette users were at higher risk of obstructive sleep apnea [36]. Night-time cravings for nicotine further exacerbate sleep disturbances, compounding the difficulty of maintaining restful sleep throughout the night. These combined effects highlight the complex interplay between nicotine dependence, respiratory health, and sleep quality, suggesting that interventions aimed at reducing nicotine consumption in young adults and managing pulmonary symptoms could improve sleep outcomes in this population.

Our study has both strengths and limitations. A notable strength of our study includes the use of a nationally representative dataset from the National Health Interview Survey (NHIS). This large, diverse data set allows for robust statistical analysis and increases the reliability of our results. A stratified approach focusing on a critical sub-population of young adults, who are particularly vulnerable to both smoking behaviors and sleep disturbances. Furthermore, we meticulously adjusted for a range of socio-demographic covariates, such as age, gender, and socioeconomic status. We also adjusted for alcohol use, which has a noted adverse effect on sleep quality [37]. Likewise, we adjusted for asthma and chronic respiratory conditions in our multivariable analysis. Despite its strengths, our study has several limitations. The cross-sectional design of the NHIS survey limits our ability to establish causation between the dual use of cigarettes and e-cigarettes and poor sleep outcomes. We also lack detailed information on the type and brand of products, which can significantly impact nicotine delivery and impact on sleep. While we controlled for alcohol use, we did not account for other substances such as cannabis and cocaine, which are linked to dual use and sleep disturbance [32]. Additionally, behavioral factors affecting sleep, such as screen time, diet, physical activity, and occupational status, were also not included in our analysis [38].

Other limitations include the absence of daily usage metrics for combustible and e-cigarette users and the lack of obstructive sleep apnea (OSA)-related variables, which hindered risk assessment among overweight or obese participants. The dataset also did not include insomnia-related variables, preventing us from evaluating its potential impact as a comorbidity. These unmeasured factors may introduce residual confounding and bias our results. Future research should aim to address these limitations by utilizing longitudinal data, gathering detailed information on nicotine use, and controlling for a broader range of lifestyle and behavioral factors. Incorporating OSA screening and insomnia assessments, such as the Insomnia Severity Index (ISI), would also enhance our understanding of the relationship between sleep disorders and smoking behaviors.

## Conclusion

This study identifies significant associations between conventional cigarette and e-cigarette use with sleep outcomes in young adults aged 18-35 years. Dual users exhibited poorer sleep quality, higher rates of sleep medication use, and more frequent sleep disturbances compared to non-users and single-product users. Further research is warranted to explore the underlying mechanisms of these associations and to develop targeted interventions to address the sleep effects of dual use in young adults. Additionally, future research should also focus on collecting and analyzing detailed information on the types and brands of both electronic and combustible cigarettes, as these factors may significantly impact nicotine delivery and sleep health.
